# Oral suction capacity in breastfeeding vs. bottle feeding. A systematic review

**DOI:** 10.3389/fped.2025.1646225

**Published:** 2025-11-07

**Authors:** Pilar Andrea Guzmán Sánchez, María Margarita Arciniegas Morera, Laura Cárdenas Jaramillo, Mariana Larrahondo Gómez, Cyntia Paola Lambis Cano, Luz Adriana Meneses-Urrea

**Affiliations:** 1 “Speech Therapy and Psychology” Research Group (Recognized by Colciencias), Universidad Santiago de Cali, Cali, Colombia; 2 Department of Phonoaudiology, Universidad Santiago de Cali, Cali, Colombia; 3 Universidad Santiago de Cali, Cali, Colombia; 4 Research Group “Health Care (Recognized by Colciencias)”, Universidad Santiago de Cali, Cali, Colombia; 5 Department of Nursing, Universidad Santiago de Cali, Cali, Colombia

**Keywords:** infant, newborn, breastfeeding, bottle feeding, sucking behavior

## Abstract

**Objectives:**

This study aims to ascertain whether breastfed infants exhibit superior oral sucking abilities compared with bottle-fed infants.

**Methods:**

A systematic review was conducted, encompassing four databases associated with professional health practices: Web of Science, Scopus, PubMed, and Dimensions. The review encompassed articles published from 2010 onward, and included children up to the age of 2 y under normotypic conditions. The search was conducted using a query constructed from keywords that considered MeSH terms, and the query was applied in all databases. The systematic review was performed following PRISMA 2020 guidelines, and the methodological quality was assessed using the MINORS scale.

**Results:**

Behaviors related to maternal suckling, such as position, mother-child bonding, adequacy of suckling, baby responses, and anatomy, showed that the group of bottle-fed babies performed poorly in the five behaviors analyzed (*P* < 0.001), with suckling behavior standing out.

**Conclusion:**

The mechanics of sucking exhibits differences in oral motor behavior between bottle-fed and breastfed infants, favoring the latter group. However, these disparities appear inconsequential in children receiving mixed feeding.

## 1 Introduction

The World Health Organization recommends exclusive breastfeeding during the first six months of an infant's life, and its continuation alongside complementary foods until at least two years of age, given that breast milk provides optimal nutrition for infants by supplying water, fats, proteins, vitamins, and essential nutrients that support cognitive and psychological development, growth, and weight gain during early childhood (1).

Sucking can be classified as non-nutritive and nutritive. The former emerges at approximately 18–24 weeks of gestation and contributes to the development of feeding skills, whereas the latter involves coordinated swallowing and occurs at approximately 32 weeks of gestation, maturing by birth to allow breastfeeding (2).

The sucking pattern is important for successful infant feeding as it allows the baby to achieve oral–motor skills. These skills depend on the integration and synchronization of the structures involved in this process, such as the lips, cheeks, suction pads, tongue, and palate. These structures extract the food content and propel it from the oral cavity to initiate the swallowing process, an action that begins the digestion and the subsequent absorption of nutrients (3).

After birth, nutritive sucking unfolds as a triadic process comprising sucking, swallowing, and breathing phases and is known as the sucking triad. The sucking process begins with the recognition and grasping of the nipple, which is facilitated by the contractions of the periorbicular muscles of the infant's lips in response to the visual sensory stimulation provided by nipple hyperpigmentation and the prominent delineation of Montgomery's glands. Once the nipple enters the oral cavity, anteroposterior mandibular movements occur, generating positive pressure, followed by negative pressure due to mandibular retraction mediated by the contraction of the suprahyoid muscles. This, together with the tongue's backward motion, transports the food content from the oral cavity to the pharynx, initiating the swallowing phase. The superior constrictor of the pharynx contracts, which favors the elevation of the palatal velum to occlude the upper airway. Simultaneously, the tongue moves the bolus toward the hypopharynx, causing the inhibition of breathing, which is known as swallowing apnea. For the third phase of the triad to be effective, breathing, the expression of suction, and swallowing must work concertedly (3).

Therefore, breastfeeding promotes healthy growth, adequate child development and decreases the occurrence of chronic diseases. According to Unicef in 2020, the rate of exclusive breastfeeding during the first six months of life in Latin America and the Caribbean was 37.3%, below the world average of 43.8%. Mesoamerica has made significant progress in this regard, from 21.6% in 2012 to 31.9% in 2020; although an improvement is evident, it is still below the global average of 44%, and below the figures of the Sustainable Development Goals (between 50% and 70%). In the Caribbean, on the other hand, exclusive breastfeeding during the first six months of life decreased between 2012 and 2020, to a rate of 27.3%. In South America, the rate was closer to the global average, at 42% (4).

The aforementioned data highlights that the use of the bottle tends to be more prevalent than breastfeeding practices. The bottle, an instrument employed for infant feeding aimed to simulate the maternal breast, exhibits diverse teat configurations on the market. However, designing a teat that fully aligns with anatomical characteristics, while preserving the function of the suction and the muscular activity of all the structures involved in the milk-extraction, remains a challenge. In addition to this, different studies, such as “Effects of the Duration of Breastfeeding, Bottle Feeding and Non-Nutritive Sucking Habits on the Occlusal Characteristics of the Primary Dentition” have indicated that the frequent use of bottles carries the risk of inducing oral parafunctional habits that alter craniofacial and oral muscular structures. This disruption subsequently compromises both initial feeding processes and the motor aspects of speech development (5). Therefore, this secondary study review aims to ascertain whether breastfed infants possess greater oral sucking abilities than bottle-fed infants.

## 2 Methodology

The study was conducted following a methodological design of systematic review, exploring literature and publications within scientific databases in adherence to the PRISMA 2020 guidelines and principles. Additionally, the research was registered in PROSPERO (288056).

### 2.1 Search strategy

The search strategy is based on MeSH terms and Boolean operators with which the following formula is defined [(infant OR “infant newborn” OR newborn) AND breastfeeding AND bottle AND feeding AND sucking behavior], which is replicated in four sources related to health disciplines: Dimensions, Pubmed, Web of science and Scopus. The search was carried out as of 2010, selecting papers written in English, Spanish and Portuguese.

### 2.2 Inclusion and exclusion criteria

Inclusion criteria were established in accordance with the PECO (Patient or Population, Exposure, Comparison, Outcome) strategy. Articles were required to satisfy the following criteria:

### 2.3 Population

The population samples of the selected articles were to study children aged 0–2 y, devoid of any history or presence of anatomical, physiological, or cognitive abnormalities. This selection was made based on the critical developmental periods of oral motor acquisition, considering the breastfeeding or bottle-feeding phase.

### 2.4 Exposure

Articles employing longitudinal or cross-sectional interventions were selected, in which the oral behavior of children within the sample was assessed and tracked using instruments such as protocols, evaluation instruments, muscle assessment equipment, and structured or semistructured interviews for data extraction.

### 2.5 Comparison

Documents that reported sucking behavior during bottle feeding and maternal sucking were included.

## 3 Results

**Figure 1:**
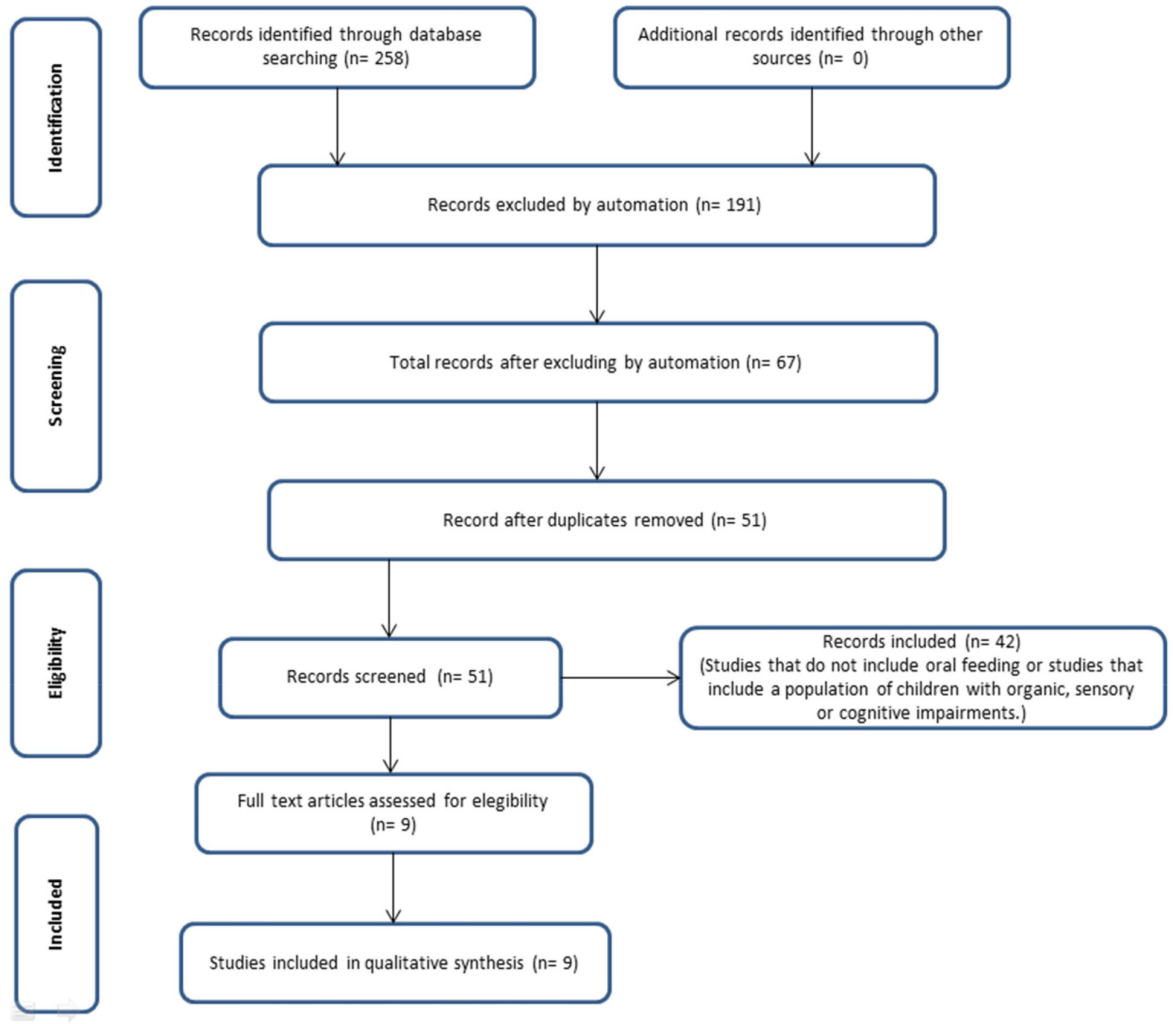
Flow chart. Self-formulated following PRISMA 2020 guidelines.

During the search and selection process, 258 records were identified through electronic database searches, with no additional sources retrieved. After removing irrelevant entries, 67 articles remained for evaluation. During screening, 191 records were excluded through automated processes, and 51 records were retained after duplicate removal. In the eligibility phase, all 51 records were reviewed, and 9 full-text articles met the inclusion criteria. Ultimately, 9 studies were incorporated into the qualitative synthesis, while 42 were excluded for not meeting the inclusion criteria, primarily due to the absence of oral feeding assessment or inclusion of children with specific disabilities (Figure 1). The articles were required to include a direct comparison between infants fed with breast milk and those fed with bottles or using pacifiers. This comparison should elucidate their impacts on oral–motor development, stomatognathic functions, or even craniofacial structures. The results could be described via statistical analyses using dependent and independent variables of oral motor behavior, information about the mother, data on the infant, and behavior during sucking. This would lead to the formulation of a quantitative or qualitative hypothesis in line with the objective of the study.

Studies were excluded if:

- The article was published in a language other than English, Spanish, or Portuguese. Furthermore, articles published before the year 2010 were disregarded to ensure the collection of recent information.
- The articles that did not allow full download

### 3.1 Assessment of methodological quality

The methodological quality of the chosen articles identified using the search strategy was appraised using the MINORS bias scale. This scale comprises 12 predetermined principles or evaluated questions, with the last 4 questions applied solely to comparative studies. This scale allowed the development of an objective evaluation by means of a score of 0 - 1 - 2 determining the quality of the information presented in the articles, with 0 signifying not informed, 1 denoting informed but inadequately informed, and 2 indicating adequately informed. The scale establishes a score based on the total number of items evaluated, resulting in a maximum score of 24 points. Therefore, an acceptable score is considered when half of the total evaluation points are obtained.

### 3.2 Methodological quality

For the analysis of the methodological quality developed from the MINORS scale, the studies were categorized based on their design. For the 7 articles with an observational cross-sectional design, the scores ranged from 9 to a maximum of 16. The comparative cross-sectional study garnered a score of 22, whereas the longitudinal study achieved a score of 14. The bias evaluation results indicated that the articles incorporated in the review met the required methodological quality, given that the score for each one exceeded 6 (see Table 1).

**Table 1:** Methodological quality of the studies (MINORS scale).

Study	1 clearly defined objective	2 consecutive inclusion of patients	3 information collected retrospectively	4 target-oriented evaluations	5 neutral evaluations	6 follow-up phase consistent with the objective	7 dropout rate during follow-up <5%	8 prospective estimate of sample size	9 appropriate control group	10 simultaneous groups	11 homogeneous party groups	12 appropriate statistical analysis	SCORE
(6)	2	2	2	2	2	0	2	2					14/24
(12)	2	2	2	2	2	2	2	2					16/24
(13)	2	2	2	2	2	2	2	2					16/24
(9)	2	2	2	2	0	2	2	2					14/24
(10)	2	2	2	2	2	2	2	2					16/24
(9)	2	2	2	2	0	2	0	2					12/24
(15)	2	2	2	2	0	2	2	2	2	1	2	2	22/24
(16)	2	2	0	2	0	0	1	2					9/24
(17)	2	2	2	2	0	2	1	2					13/24

**Figure 2:**
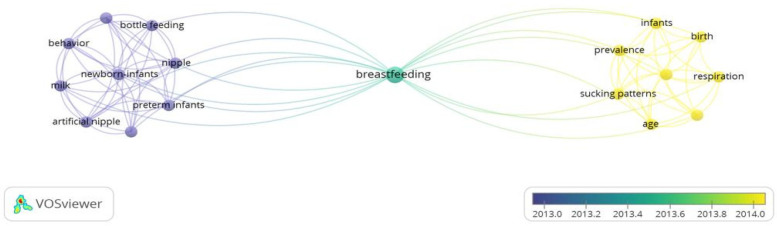
Keywords (VOS VIEWER).

As depicted in Figure 2 generated via the VOSviewer v.1.6.18 tool, the co-occurrence of the most relevant terms identified in the selected articles from the Web of Science database was established. The nodes represented the keywords, and their size was related to their frequency. Thus, it was concluded that “breastfeeding” was a term that was related to the terms of age, sucking patterns, behavior, breathing, infants, birth, prevalence, bottle feeding, newborns, nipple, milk, artificial teat, and premature. Most of these terms were considered in the search formula constructed from the PECO strategy. It was also observed that the publications with the greatest impact for this research were included within the timeframe of 2013–2014.

The most significant variables identified in the articles reviewed (Table 2) were related to the sociodemographic data of the population, including children and mothers, as well as prenatal, perinatal, and postnatal aspects. Likewise, variables related to the duration of bottle feeding and breastfeeding were identified, together with the percentage of each feeding practice. Finally, variables directly related to the sucking behavior and the infant's feedings per min ratio were considered. The distribution of the articles corresponding to each variable is shown in Table 2.

**Table 2:** Prevalence of the most significant variables.

Variable and research relevance	
○ Infant's age	(*n* = 7)
○ Infant's sex	(*n* = 7)
○ Maternal age	(*n* = 2)
○ Maternal marital status	(*n* = 1)
○ Maternal occupation	(*n* = 1)
○ Maternal educational level	(*n* = 2)
○ Gestational age (weeks, mean ± SD)	(*n* = 5)
○ Birth weight (g, mean ± SD)	(*n* = 5)
○ Infant's age at initial feeding assessment (days, mean ± SD)	(*n* = 2)
○ Duration of breastfeeding	(*n* = 2)
○ Duration of bottle feeding	(*n* = 1)
○ Proportional distribution of each	(*n* = 2)
○ Feeding practice during the first 6 Mo	
○ Bottle feeding	(*n* = 2)
○ Use of pacifier or teat	(*n* = 2)
○ Suction behavior	(*n* = 2)
○ Number of suctions per minute	(*n* = 1)
○ APGAR score	(*n* = 2)
○ Received prenatal care	(*n* = 1)
○ Type of delivery	(*n* = 1)
○ Pregnancy type	(*n* = 1)

#### 3.2.1 Sociodemographic data of the population included in the studies

The articles incorporated in the study indicated a balanced distribution of male and female infants, spanning an age spectrum from newborn to 48 months. In terms of maternal information, factors such as age, marital status, occupation, and educational background were considered. Consequently, it was identified that the mothers participating in the research process were aged between 19 and 30 y, with 70.2% having a partner and 57.4% registered as unemployed. With regard to schooling, the levels of education varied from basic education to postgraduate studies.

#### 3.2.2 Prenatal, perinatal, and postnatal characteristics

Variables related to gestational aspects and prenatal care during pregnancy, including baby-specific attributes, such as gestational age, birth weight, and APGAR (Activity, Pulse, Grimace, Appearance, Respiration) results, emerged as noteworthy in the examined articles. In 99.3% of the cases, the most frequently reported gestational age was 39 weeks with the inclusion of prenatal medical care. Regarding delivery methods, a comparable distribution was observed, with vaginal delivery in 50.8% of the cases and cesarean delivery in 49.2% of the cases. The birth weight was >2500 g, which is considered a normal weight for newborns at term (6).

#### 3.2.3 Duration of breastfeeding and/or bottle feeding

In a specific study encompassing 734 children, the durations of both breastfeeding and bottle feeding were outlined. Within the first 6 months, exclusive breastfeeding was reported in 22.9% of the infants and 14.2% were exclusively bottle-fed. The predominant feeding approach was mixed and accounted for 62.9% of the cases. After 6 months and up to 18 months of age, bottle feeding was administered to 41.6% of the children while 59.1% continued with breastfeeding (5).

#### 3.2.4 Sucking behavior in breastfeeding and bottle feeding

The study entitled “Mechanics of sucking: comparison between bottle feeding and breastfeeding” established that infants aged 21–28 d that were exclusively bottle-fed exhibited fewer sucks but the same number of pauses, which were longer than those in breastfed infants. In mixed feeding, bottle-fed infants vs. breastfed infants presented no significant differences in the number of suctions, with shorter pauses compared with those that were exclusively bottle-fed, both in neonates and younger infants. In the mixed feeding group, neonates averaged 5.83 ± 1.93 suctions per d, while younger infants (up to 5 months) averaged 4.42 ± 1.67. Bottle-fed and breastfed infants shared similar oral movements, although differences were noted in sucking mechanics, i.e., number of sucks, pauses per minute, and duration of sucking. However, these differences were deemed nonsignificant, leading to the conclusion that mixed-fed infants develop a different sucking pattern that does not affect either feeding method (7).

Upon analyzing the nine selected articles (Table 3), we observed a pattern demonstrating that prolonged bottle use leads to a series of consequences not only in relation to sucking pattern behavior but also in anatomical and functional alterations during orofacial development, specifically in the posture and muscle dynamics of the lips and tongue. A cross-sectional study involving 427 infants and addressing behaviors such as position, mother–child binomial affectivity, adequacy of sucking, baby's responses, and mother's anatomy showed poor performance in the bottle-fed group across all five behaviors analyzed (*p* < 0.001), with a particular emphasis on the sucking behavior (8).

**Table 3:** Methodological analysis of the articles.

Author	Target	Participants	Exhibition	Variables	Significant results (p*)	Descriptive results
(8)	To investigate the association between pacifier use and bottle feeding and unfavorable breastfeeding behaviors	Population: Newborns and mothers. Sample: 427	Bottle/teat: Not specified Breastfeeding: Not specified	- Mother/child position - Binomial responses - Suction behavior - Breast anatomy - Affectivity - Baby's age - Sex of the baby - Maternal age - Mother's marital status Mother's occupation - Family income - First child - Type of delivery - Prenatal care received - Place of delivery - Information received on breastfeeding at the pre and/or postnatal consultation - Bottle feeding - Use of the pacifier	Breastfeeding showed better behavior, favorable for: - Position mother/infant: <0.001 - Affectivity: <0.001 - Suction behavior: <0.001 - Infant response: <0.001 - Breast anatomy: <0.001	Not specified
(7)	To evaluate the mechanics of feeding movements in exclusive breastfeeding, bottle feeding, and mixed feeding	Population: infants and newborns. Sample: Not specified	Bottle/teat: Not specified Breastfeeding: Not specified	- Age - Type of feeding. - Mother's age. - Type of pregnancy - Sex of the infant - Gestational age, week, mean - Weight at birth - Infant age at first feeding measurement, days, mean. - Feeds per day in the last week - Time since last feed - Number of suctions per minute - Number of pauses per minute - Duration of pauses per minute	Not specified	Mixed or bottle feeding showed significant changes compared with breastfeeding: - Less suction movements - Shorter suction duration - Weaker suction pattern
(6)	To analyze the electrical activity of the masseter muscle via surface electromyography during sucking in term newborns by comparing breastfeeding, bottle feeding, and cup feeding	Population: Newborns Sample: 15	Bottle/teat: Not specified Breastfeeding: Not specified	- Gestational age. - Birth weight (grams) - APGAR score	Improved masseter electrical activity with breastfeeding: *p* = 0.003	Not specified
(4)	To investigate sucking habits, nighttime mouth breathing, and the relationship between these factors and malocclusion	Population: mothers and children from 0 to 30 months of age Sample: 80 mother–child pairs	Bottle/teat: Not specified Breastfeeding: Not specified	- Finger suction - Pacifier sucking - Bottle feeding - Breastfeeding - Nocturnal mouth breathing	Lack of breastfeeding or early weaning can generate: - Overjet (12–18 mo): <0.0001 - Overjet (30 months): 0.001 - Open bite (12 mo): 0.0002 - Open bite (18 mo): 0.001 - Open bite (30 months): 0.01	Not specified
(5)	To evaluate the effects of breastfeeding duration, bottle feeding duration, and non-nutritive sucking habits on the occlusal characteristics of the primary dentition in 3- to 6-year-old children in Beijing city	Population: Children Sample: 734	Bottle/teat: - 6–18 mo: 41.6% - >18 mo: 58.4% Breastfeeding: - Never breastfed: 13.8% Breastfed from 1 to 6 mo: 27.1% - - Breastfed for >6 mo: 59.1%	- Age - Child's sex - Mother's level of education - Non-nutritive sucking habits - Duration of breastfeeding and bottle feeding - Percentage of each feeding practice in first 6 mo	Short duration of breastfeeding: never or <6 mo: - Posterior crossbite: 0.031 - Suction to the pacifier: 0.0002 - Digital suction: <0.001	Not specified
(10)	To investigate the influence of oral habits and breastfeeding on children's oral skills	Population: Infants 9 mo of age. Sample: 125	Bottle/teat: Not specified Breastfeeding: Not specified	- Weight - Length and gestational age at birth - APGAR score - Current weight	Oral habits significantly affecting proper sucking skills: - Nipple: <0.001 - Foreign objects: <0.02	Not specified
(9)	To compare orofacial movement and mouth angle during breastfeeding and bottle feeding in normal infants	Population: Infants Sample:12	Bottle/teat: Not specified Breastfeeding: Not specified	- Milk intake - Mouth angle - Sex of the baby - Throat region - Type of feeding	Breastfeeding favors the opening of the mouth: - Mouth angle: <0.001	Not specified
(12)	To measure intraoral pressure and perioral movement in infants during breastfeeding and experimental teat feeding. The nipple has a wide base, a firm shaft, and a valve at the base so that milk flows only when the infant maintains pressure.	Population: Infants 1–8 mo of age. Sample: 20	Bottle/teat: Not specified Breastfeeding: Exclusive breastfeeding: 48.4%	- Mouth angle - Milk intake - Intraoral pressure - Number of suctions - Duration of suction	Not specified	The analysis of infant sucking patterns found no significant differences between the two feeding methods. Therefore, these findings suggest that the infant has a similar number of sucking cycles per burst and the same duration of sucking bursts when feeding from the breast and from the experimental nipple.
(11)	To determine the prevalence of mouth breathing and to associate breastfeeding history with infant breathing patterns	Population: boys and girls 30–48 mo of age. Sample: 252	Bottle/teat: Not specified Breastfeeding: Not specified	- Age - Sex - Type of breastfeeding - Respiratory pattern	Longer duration of breastfeeding significantly decreases the presence of oral respiration: Bottle feeding: <0.001 - Non-nutritive suction: 0.009	Not specified

* indicates a statistically significant result.

An electromyographic analysis conducted in these studies demonstrated greater effectiveness of masseter muscle activity during breastfeeding compared with bottle feeding (*p* = 0.003, ANOVA) (6). In addition to muscle activity, the relationship of orofacial movement and mouth angle during breastfeeding and bottle feeding was also identified in normotypic infants, where breastfeeding favored mouth opening (*p* < 0.001) (9).

Four of the studies included in the review also described variables in relation to oral habits and occlusal characteristics. As described by Moimaz, oral habits lead to or predispose to occlusal characteristics, with alterations in the vertical and horizontal planes (overjet–overbite). This was associated with the type of feeding of the children included in the study, where prolonged bottle feeding at 12, 18, and 30 mo was associated with some type of malocclusion, and together with the use of pacifiers, was associated with overjet, open bite, horizontal overbite, and posterior crossbite (6). Likewise, Chen showed in 2015 that children bottle-fed for over 18 months had a 1.45-fold increased risk of non-mesial step occlusion and a 1.43-fold increased risk of class II canine relationship (10). Lopes exposed an association between bottle feeding and the presence of non-nutritive sucking habits (*p* < 0.001), predisposing children to the acquisition of an oral breathing pattern (11). Maciel described the negative influence of pacifier use on oral sucking skills in children up to 9 months of age, particularly in relation to breastfeeding (OR 3.1; 95% CI 1.2–8.3) (10).

In response to the implications of bottle use, a study explored the use of an experimental teat (ET) as an alternative. In this regard, comparison of the behavior associated with breastfeeding and the teat showed no significant differences in perioral movements and sucking behaviors with the use of the ET. Hence, it was concluded that the ET might mitigate breastfeeding issues related to bottle use. However, further investigation is warranted to examine its potential to mitigate adverse behaviors arising from prolonged use (12).

## 4 Discussion

Sucking is a reflex activity in the newborn that becomes integrated within the first 4 mo of life and undergoes specialization following oral–motor activity (13). As the infant develops, it culminates in fine and dissociated movements of the oral cavity structures, allowing the acceptance of more solid foods after 6 mo and the stabilization of the structures in speech function. The sucking performed by the infant when breastfeeding has been recognized as a natural activity that requires the coordination of vegetative functions and its structures for the extraction of human milk (14). The oral–motor behavior during infant feeding shows differences between sucking associated with breastfeeding and sucking associated with bottle feeding. In some cases, the confusion that may arise with the nipple and teat compromises the permanence of breastfeeding, being necessary to recognize from scientific evidence if those babies who are exposed to a bottle have the same oral–motor behavior as children who are breastfed (15).

Sucking can be quantified in terms of number of suctions, intraoral force, muscle activity, swallowing pauses, and pause duration (7). According to Franca et al., identified reduced masseter muscle activity in the bottle-fed infant group compared with exclusively breastfed infants, indicating enhanced functional effectiveness of oral opening in the latter (6). In relation to the sucking mechanics determined by the number of suctions, pauses and duration, there are differences between bottle feeding and breastfeeding. Favorable outcomes tend to manifest in the latter; however, in instances of shared feeding (mixed feeding), these differences become negligible (16).

The study conducted by López indicated that breastfeeding contributes significantly to the maturation of the stomatognathic system and craniofacial growth, demonstrating a direct association with mandibular stabilization and facial harmony in breastfed children. Conversely, the study remains inconclusive regarding the benefits of mixed feeding. This correlation is pivotal because at 6–8 month of age, children achieve the correct position in relation to the upper jaw, preventing malocclusions related to horizontal overbite (17). Similarly, Sakalidis et al. showed similar responses during maternal sucking, indicating a more favorable oral–motor behavior due to enhanced control over sucking bursts and increased pauses as children age (16).

Currently, despite the promotion of breastfeeding as the feeding method of choice, global rates remain suboptimal. Consequently, the adoption of alternative feeding techniques and the use of pacifiers can precipitate bite abnormalities (18). In addition, harmful oral habits related to sucking may arise, along with poor development of the mandibular structure due to restricted muscle functionality (19). In this context, this study contributes to the scientific evidence supporting breastfeeding as a protective factor against facial musculoskeletal anomalies (20) and as a facilitator in the acquisition of refined oromotor skills essential for speech (4, 7, 17). The accumulated evidence demonstrates the multifaceted benefits of breastfeeding, including its impact on sucking mechanics, craniofacial development, and stomatognathic system functions.

Among the limitations of the study, it was identified that most existing research focuses primarily on the nutritional benefits of breastfeeding, while few experimental studies examine its specific effects on orofacial growth and function. Therefore, additional experimental research is needed to provide further evidence on the differential benefits of breastfeeding vs. the use of pacifiers in the development of the oral cavity.

## References

[1] World Health Organization. Exclusive Breastfeeding. Geneva: World Health Organization (2011). Available online at: https://apps.who.int/nutrition/topics/exclusive_breastfeeding/es/index.html (Accessed May 2024).

[2] La Orden Izquierdo E Salcedo Lobato E Cuadrado Pérez I Herráez Sánchez MS Cabanillas Vilaplana L. Delay in the acquisition of sucking-swallowing-breathing in the preterm; efects of early stimulation. Nutr Hosp. (2012) 1126:112. 10.3305/nh.2012.27.4.5848 23165551

[3] Rendón M Serrano G. Physiology of nutritive sucking in newborns and infants. Bol Med Hosp Infant Mex. (2011) 68:319–27. Available online at: scielo.org.mx/pdf/bmim/v68n4/en_v68n4a11.pdf

[4] Moimaz SAS Garbin AJT Lima AMC Lolli LF Saliba O Garbin CAS. Longitudinal study of habits leading to malocclusion development in childhood. BMC Oral Health. (2014) 14:96. 10.1186/1472-6831-14-96 25091288 PMC4126276

[5] Chen X Xia B Ge L. Effects of breast-feeding duration, bottle-feeding duration and non-nutritive sucking habits on the occlusal characteristics of primary dentition. BMC Pediatr. (2015) 15:46. 10.1186/s12887-015-0364-1 25895651 PMC4422261

[6] França EC Sousa CB Aragão LC Costa LR. Electromyographic analysis of masseter muscle in newborns during suction in breast, bottle or cup feeding. BMC Pregnancy Childbirth. (2014) 14:154. 10.1186/1471-2393-14-154 24885762 PMC4014087

[7] Moral Á Bolibar I Seguranyes G Ustrell JM Sebastiá G Barba CM Mechanics of sucking: comparison between bottle feeding and breastfeeding. Matronas Prof. (2011) 12:9–17. 10.1186/1471-2431-10-6 PMC2837866 20149217

[8] Batista CLCC Ribeiro VS do Nascimento MDSBB Rodrigues VP. Association between pacifier use and bottle-feeding and unfavorable behaviors during breastfeeding. J Pediatr (Rio J). (2018) 94:596–601. 10.1016/j.jped.2017.10.005 29136496

[9] Aizawa M Mizuno K Tamura M. Neonatal sucking behavior: comparison of perioral movement during breast-feeding and bottle feeding. Pediatr Int. (2010) 52:104–8. 10.1111/j.1442-200X.2009.02914.x 19552641

[10] Maciel AR. Aleitamento materno e sua infl uência nas habilidades orais de crianças infl uence of breastfeeding on children ‘ s oral skills. Rev Saúde Pública. (2013) 47:37–43. 10.1590/S0034-89102013000100006 23703128

[11] Lopes TSP Moura LFAD Lima MCMP. Association between breastfeeding and breathing pattern in children: a sectional study. J Pediatr (Rio J). (2014) 90:396–402. 10.1016/j.jped.2013.12.011 24703820

[12] Segami Y Mizuno K Taki M Itabashi K. Perioral movements and sucking pattern during bottle feeding with a new experimental teat are similar to breastfeeding. J Perinatol. (2013) 33:319–23. 10.1038/jp.2012.113 22975983

[13] Morales F. Assessment and management of sucking-swallowing difficulties in newborns and infants without neurmomuscular disease. Neumol Pediátrica. (2019) 14:138–44. 10.51451/np.v14i3.104

[14] Durán-Gutiérrez A Rodríguez-Weber M de la Teja-Ángeles E Zebadúa-Penagos M. Succión, deglución, masticación y sentido del gusto prenatales: desarrollo sensorial temprano de la boca. Acta Pediatr Mex. (2012) 33:137–41. Available online at: chrome-extension://efaidnbmnnnibpcajpcglclefindmkaj/https://www.medigraphic.com/pdfs/actpedmex/apm-2012/apm123g.pdf

[15] Espinal G Hoyos LM Ramos C Salcedo B Arango A Beatriz Aguirre LJP. Comparacion electromiografica de musculos suprahioideos, maseteros y orbicular de los labios en niños y niñas de 1 a 5 meses de edad, alimentados con lactancia materna o biberon. 2009. Rev Fac Odontol Univ Antioq. (2021) 12(2):4. Available online at: https://dialnet.unirioja.es/servlet/articulo?codigo=9402400

[16] Sakalidis VS Kent JC Garbin CP Hepworth AR Peter E Hartmann DTG. Longitudinal changes in suck-swallow-breathe, oxygen saturation, and heart rate patterns in term breastfeeding infants. J Hum Lact. (2013) 29(2):236–45. 10.1177/0890334412474864 23492760

[17] López Rodríguez YN. Infant oral motor function as a stimulus for craniofacial growth. Univ Odontol. (2016) 35:1–37. 10.11144/Javeriana.uo35-74.fmol

[18] Thomaz EB Alves CM Gomes E Silva LF Ribeiro de Almeida CC Soares de Britto E Alves MT Breastfeeding versus bottle feeding on malocclusion in children: a meta-analysis study. J Hum Lact. (2018) 34(4):768–88. 10.1177/0890334418755689 29596751

[19] Goovaerts S El Sergani AM Lee MK Shaffer JR Claes P Weinberg SM. The impact of breastfeeding on facial appearance in adolescent children. PLoS One. (2024) 19(9):1–9. 10.1371/journal.pone.0310538 PMC11407646 39288146

[20] Pereira Lopes TS Branco Lima CC Cerqueira Silva RN Almeida de Deus Moura LF Moura de Lima MD Pinheiro Lima MC. Association between duration of breastfeeding and malocclusion in primary dentition in Brazil. J Dent Child. (2019) 86(1):17–23. 10.1186/s12887-015-0364-1 30992097

